# A human IgM enriched immunoglobulin preparation, Pentaglobin, reverses autoimmune diabetes without immune suppression in NOD mice

**DOI:** 10.1038/s41598-022-15676-8

**Published:** 2022-07-11

**Authors:** Christopher S. Wilson, Emilee M. Hoopes, Alexander C. Falk, Daniel J. Moore

**Affiliations:** 1 Department of Pediatrics, Ian Burr Division of Endocrinology and Diabetes, Vanderbilt University Medical Center, Nashville, TN USA; 2 Department of Pathology, Microbiology, and Immunology, Vanderbilt University Medical Center, 7415 MRB4, 2213 Garland Avenue, Nashville, TN USA; 3 Division of Endocrinology, Department of Pediatrics, Vanderbilt University Medical Center, 7415 MRB4, 2213 Garland Avenue, Nashville, TN 37232 USA

**Keywords:** Autoimmunity, Immunotherapy

## Abstract

The immune system of healthy individuals is capable of regulating autoimmunity through multiple mechanisms. In Type 1 Diabetes (T1D) we recently discovered natural IgM, although present at normal levels, is unable to perform its normal immunoregulatory function. Treating diabetic mice with IgM from healthy donors led to reversal of disease without immune depletion. To investigate the therapeutic potential of a human preparation of IgM, we administered an IgM-enriched preparation of immunoglobulin called Pentaglobin. Administration of Pentaglobin therapy reversed disease in diabetic NOD mice and boosted CD4 + Foxp3 + Tregs. Importantly, the impact of Pentaglobin on the immune system was limited to inhibiting beta cell destruction but was not immune depleting nor did it inhibit the immunization response to an irrelevant antigen. These findings indicate that inhibition of deleterious autoimmunity in T1D is possible while leaving protective immunity fully intact.

## Introduction

Type 1 diabetes (T1D) is an autoimmune disease that results from the collaboration of T and B lymphocytes to drive the destruction of beta cells of the islet^1,2^. Destruction of beta cells leads to the need for continuous insulin therapy that, while life sustaining, comes with the potential for multiple co-morbidities even with tight glucose control^3^. Curative therapy is sorely needed to curb the risk that treatment with insulin poses to patients with T1D. To this end, immunologic interventions have been attempted clinically to reverse or prevent T1D. While some interventions provide modest retention of C-peptide, none are curative, and each currently appears to require repeated therapy to prevent disease progression, which will result in immune suppression ranging from intermittent to continuous^4–7^. With the emergence of the global COVID-19 pandemic, which poses an increased threat to those with T1D, it is clear that the current intervention paradigm in T1D that utilizes therapies that impede or suppress autoimmunity at the expense of normal immunity is a significant risk to the community^8^.

The homeostatic maintenance of the immune compartment is crucial for normal immune function. Studies have demonstrated that abnormal immune homeostasis, including lymphopenia, encourages the emergence of autoreactive cell subsets that hasten autoimmunity. In T1D there is evidence of abnormal immune homeostasis in both animal models and humans, including altered T cell and B cell development, altered cell subsets, and production of autoantibodies^9–11^. We have previously demonstrated the circulating IgM from healthy donors has the capacity to restore immune homeostasis and reverse T1D without global immune depletion when administered therapeutically^12^.

To understand the therapeutic potential of human IgM, we investigated the ability of a preparation of IgM-enriched human immunoglobulins (Pentaglobin) to reverse diabetes in NOD mice. This clinically-utilized therapy has been applied as an adjuvant therapy in bacterial sepsis, but its efficacy in autoimmunity remains unknown^13,14^. We determined that Pentaglobin reverses diabetes in NOD mice and enhances Treg generation, while leaving the protective function of the immune system intact. These data indicate that immune dysfunction in T1D can be treated without immune depletion or suppression and indicate the potential for harnessing endogenous immune regulators to provide autoimmune regulation without immune suppression.

## Results

### Pentaglobin, an immunoglobulin preparation enriched in human IgM, reverses diabetes in NOD mice

**Figure 1:**
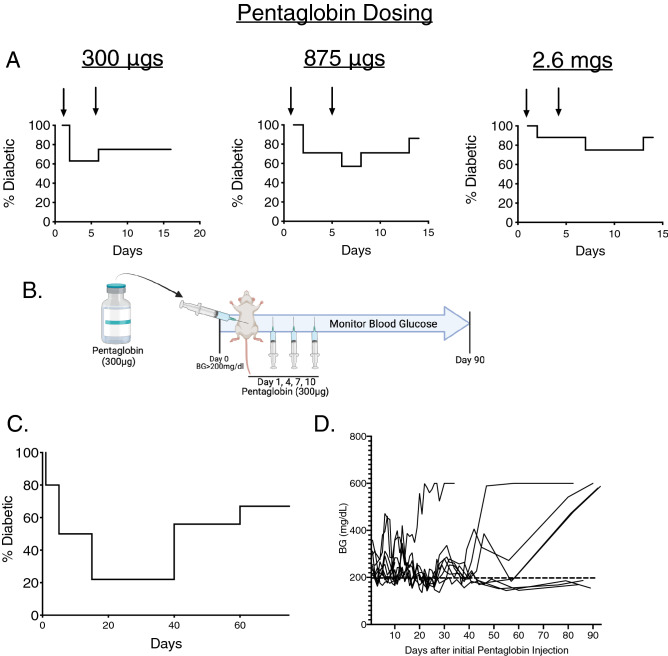
Pentaglobin reverses diabetes in NOD mice. (**A**) To determine the optimal therapeutic course for diabetes reversal by Pentaglobin, NOD mice were allowed to become diabetic as defined by two consecutive blood glucose readings between 200 and 300 mg/dL. These mice were then treated with 2 doses of 300 ug, 857 ug, or 2.6 mg of Pentaglobin on Day 1 and Day 4. (*n* = 8) The 300ug dose provided maximum effectiveness in terms of overall reversal. (**B**) 4 doses of 300ug Pentaglobin were delivered 3 days apart in newly diabetic NOD mice to achieve better reversal. (**C**) Diabetic mice given 4 injections of Pentaglobin experienced ~ 78% reversal at day 15, with approximately 33% remaining diabetes free for 90 days. (**D**) Individual blood glucose readings in NOD mice taken during Pentaglobin treatment period.

Our previous studies of purified polyclonal IgM to reverse diabetes in NOD mice utilized 2 peritoneal injections of 100ug of IgM^12^. Based on these studies, we utilized a similar reversal strategy with Pentaglobin doses containing the equivalent of 35ug, 100ug, and 300ugs of IgM, which equaled a dose of 300ug, 875ug, and 2.6mgs of total Pentaglobin (78.5% IgG 10% IgA and 11.5% IgM), respectively. NOD mice were deemed diabetic after two consecutive days of blood glucose readings between 200-300 mg/dl. After the second elevated glucose reading, mice were treated with the indicated dose of Pentaglobin on day 1 and then again 3 days after the initial dose. Mice were considered reversed after 2 consecutive days of readings below 200 mg/dl and were scored diabetic after 2 consecutive days above 200 mg/dl. Utilizing this approach, we demonstrated 40% total reversal in the 300ug and 875ug dosing but only saw ~ 20% total reversal with 2.6 mg dosing. Since the higher dosing strategies showed no improvement over the 300ug dosing, we chose the 300ug (30ug of IgM) dose to further study for diabetes reversal (Fig. 1A). We hypothesized two additional doses would provide improved reversal in these mice (Fig. 1B). This dosing strategy led to initial reversal of ~ 78% of mice enrolled in the study and ~ 33% remained diabetes-free for 90 days after the initial dose (Fig. 1C and D). The overall reversal may favor those mice with a slightly lower starting blood glucose (240mgl/dl for stably reversed at 90 days and ~ 265 mg/dl average for those that did not stably reverse) although these differences were not statistically significant (*p* = 0.5, t-test). Importantly, we observed no mouse anti-human antibodies (MAHA) in these mice (Supplemental Fig. 1A).

### Pentaglobin reversal is linked to expanded thymic B cells and Tregs

**Figure 2:**
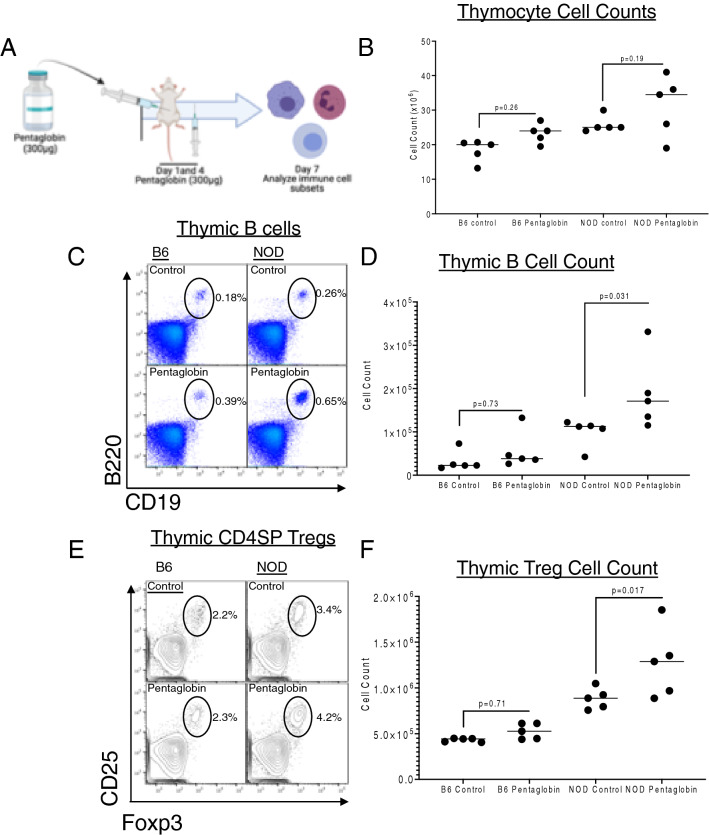
Pentaglobin expands thymic B cell and Treg numbers. (**A**) Previous studies demonstrate healthy donor IgM expands thymic B cells and Tregs to drive diabetes reversal. To determine the immunologic changes induced by Pentaglobin, we delivered 2, 300ug doses into B6 mice and prediabetic NOD mice. Mice were euthanized on day 10 and thymus and spleen isolated for flow analysis. (**B**) Total thymocyte cell counts revealed an increase in NOD mice treated with Pentaglobin. (**C**) This increase was linked to an expansion of thymic B lymphocytes in NOD mice. Quantified in (**D**). (**E**) Expansion of thymic B cells has been linked to thymic Treg expansion in other models. Flow cytometry was used to identify Foxp3 + CD25 + CD4 single-positive cells among B6 and NOD thymocytes. Thymic Tregs were expanded in NOD mice treated with Pentaglobin. Total cell counts demonstrated in (**F**).

Diabetes reversal by healthy mouse IgM in NOD mice was associated with expansion of thymic B cells and Tregs^12^. To determine whether Pentaglobin modulated the immune system in the same way as healthy mouse IgM, we treated prediabetic NOD mice with two doses of 300ug of Pentaglobin (Fig. 2A). Thymic cell counts trended toward an expansion of total thymocytes in NOD mice treated with Pentaglobin but did not reach statistical significance (Fig. 2B). Flow analysis revealed increases in total B cells in the thymus of NOD mice compared to controls (Fig. 2C right panels and quantified in D). These thymic B cells were essential for expansion of thymic Tregs in our previous studies of treatment with natural IgM. Our studies with Pentaglobin indicate that thymic B cell expansion is also accompanied by increase in total Tregs as observed in the thymus of NOD mice treated with Pentaglobin (Fig. 2E right panels and quantified in F).

### Pentaglobin expands peripheral Tregs but does not induce suppression of normal immunity

**Figure 3:**
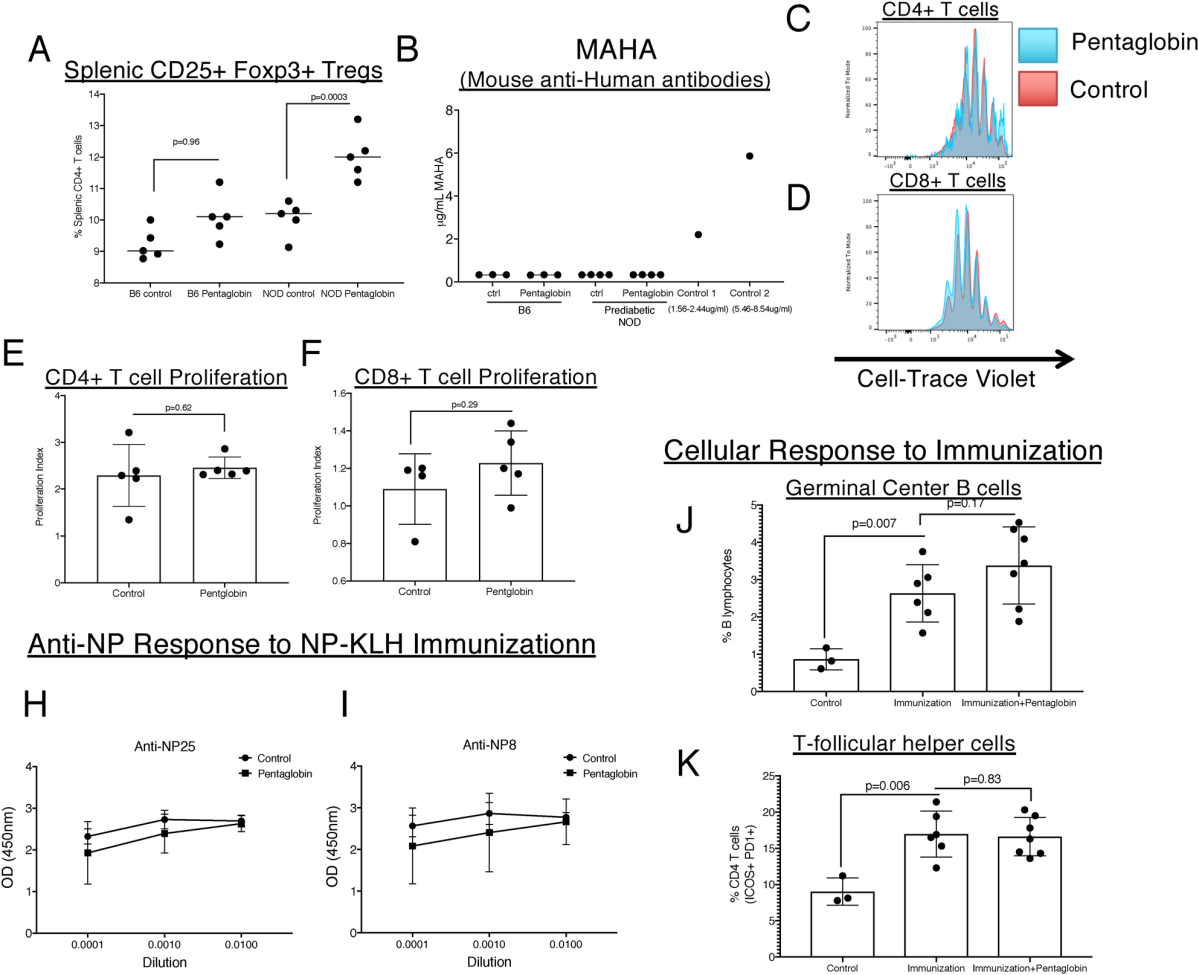
Pentaglobin fosters peripheral Treg expansion but does not induce robust immune suppression. (**A**) Analysis of the spleen revealed that Pentaglobin treatment expanded peripheral Tregs in NOD mice treated with Pentaglobin as was similarly evident in the thymus. (**B**) Although Pentaglobin did not appear to induce an immune response in treated mice, we measured the anti-human response in B6 and Prediabetic NOD mice. Using an ELISA that measures Mouse Anti-Human Antibodies (MAHA), minimal anti-human antibodies in B6 mice or NOD mice were detected before or after Pentaglobin treatment, as compared to assay controls. (**C**) To assess whether Pentaglobin was potently immunosuppressive, T cells from untreated control NOD mice and NOD mice treated with 2 doses of Pentaglobin were isolated. These cells were then labeled with a proliferation dye and cultured with anti-CD3 and anti-CD28. Proliferation of CD8 and CD4 T cells was scored by flow cytometry. No difference was observed in the proliferation of CD4 T cells. Proliferation index quantified in (**D**). (**E**) Likewise there was no difference in proliferation of CD8 T cells. Quantified in (**F**). (**H**), (**I**) To assess the B cell response, NOD mice were immunized with NP-KLH to measure T dependent B cell antibody production. Mice immunized with NP-KLH were treated with 2 doses of Pentaglobin or left untreated. Their anti-NP response was measured via ELISA. Low-affinity and high-affinity antibody responses (indicative of affinity maturation) were both intact. (**J**), (**K**) Flow analysis of the spleen from immunized mice demonstrated similar levels of GC B cells (B220 + IgM − Fas + GL7 +) and Tfh cells (CD4 + ICOS + PD − 1 +).

We observed that thymic expansion of Tregs was maintained in the splenic compartment of NOD mice treated with Pentaglobin (Fig. 3A). This observation links to the mechanism observed in NOD mice treated with mouse polyclonal IgM as Tregs were essential for stable disease reversal in those prior studies^12^. As with treated, diabetic mice, prediabetic NOD and B6 mice generated no MAHA response after Pentaglobin (Fig. 3B) As our studies indicate, repeated dosing of Pentaglobin even beyond 4 days may be important for stable reversal of T1D in a clinical setting as is the case with other current investigational interventions for T1D. Readministration of these other therapies may be limited due to their immune-depleting or suppressing nature, which may leave patients susceptible to infection, cancer, or other unknown risks that may be exacerbated as treatment courses are extended. While cell counts and flow cytometry demonstrated that Pentaglobin was not immune depleting, we further investigated whether it inhibits the proliferative capacity of protective T cells. Proliferation was similar in both CD4 and CD8 T cells in NOD mice treated with Pentaglobin or left untreated (control) (Fig. 3C–F). To assess the ability of Pentaglobin-treated NOD mice to respond to an immune challenge, mice were immunized with NP33/KLH/IFA. To assess how Pentaglobin impacts this immune response to NP, we treated NOD mice on day -1 before immunization with 300ug of Pentaglobin and again on day 7. Both the low-affinity (NP25) and the high-affinity (NP8) anti-NP IgG response were analyzed by ELISA on day 17. We detected no difference in anti-NP titers in NOD mice treated with Pentaglobin (Fig. 3H and I). Flow cytometry analysis of spleens from immunized mice demonstrated similar levels of Germinal Center B cells and T-follicular helper cells between treated and untreated mice (Fig. 3J and K).

## Discussion

This study indicates that the immune dysfunction in autoimmune T1D in NOD mice can be treated without immune suppression. This non-suppressive immune therapy was accomplished with a clinically-used, human immunoglobulin preparation that contains increased amounts of IgM (12%), Pentaglobin. This study highlights the need and opportunity to advance non-suppressive strategies that can reset immune homeostasis while maintaining protection from the threat of infectious disease in persons with T1D.

Intravenous immunoglobulin (IVIg) therapy has shown effectiveness in many immune conditions but failed to reverse clinical disease in T1D^15^. These preparations contain mostly pooled IgGs from human donors and very little IgM. While IgG is a widely utilized therapy, IgM is the most abundant, non-induced antibody found in circulation in humans and mice^16^. It is produced continuously by marginal zone B cells of the spleen and B1 B cells, located in the peritoneal cavity and bone marrow of mice^17^. IgM is the first line of defense against invading pathogens. It can regulate immune homeostasis in B and T cells, and its absence leads to perturbed immune cell development similar to that observed in NOD mice^18,19^. Previous studies indicate that purified IgM isolated from both healthy human and murine donors could prevent as well as reverse diabetes in NOD mice as well as modulate B cell homeostasis and Treg expansion^12,20^.

Pentaglobin is currently utilized as an adjuvant therapy in patients with sepsis. It has been observed in some studies to reduce mortality rates in patients with any level of sepsis severity^14^. This finding indicates that Pentaglobin can inhibit deleterious immunity, responsible for the morbidity associated with severe sepsis, while allowing ongoing clearance of infection. Our data support this model, as we demonstrated reversal of autoimmunity and enhancement of Tregs but did not observe immune suppression in T cell or B cell responses. This important property makes Pentaglobin an ideal candidate for T1D therapy where children are the primary recipients and where current therapies are largely immune depleting and suppressing making their continual delivery unfavorable.

Pentaglobin induced robust diabetes reversal in newly diabetic NOD mice. This reversal was linked to changes in thymic B cell and Treg populations. These changes were also observed with treatment of purified murine donor IgMs from healthy mice in our previous study^12^. We did not observe peripheral alterations of B cell subsets in NOD mice treated with Pentaglobin as we did with murine IgM, which may account for some differences in the observed effect on diabetes in the animal model (Supplemental 2). Nevertheless, Pentaglobin induced a modest expansion in peripheral Tregs. The reasons for these differences in immune modulation may relate to species-specific interactions. These interactions and their impact on immune function and disease demand further investigation in a clinical setting. Applying Pentaglobin therapy to humans with T1D will require monitoring increases in Tregs, especially those newly emigrated from the thymus.

The immune regulation of IgM has been linked to antigen specificity, structural changes, and glycosylation patterns^18,19,21–25^. It is possible that the human IgM bound with less affinity to immune cell subsets, due to epitope differences in cell surface molecules. While we provided the same total amount of IgM as previous studies, the amount of effective, therapeutic IgM may have differed due to the different isolation techniques. Additionally, the amount of IgG delivered concomitantly with IgM may have counteracted or masked immune changes induced by IgM alone. The higher dosage, while containing more IgM, was less effective at diabetes reversal, suggesting interactions with other components of Pentaglobin.

Maintaining normal immune function should be an important consideration when selecting an immune therapeutic for T1D or any autoimmune disease, but the degree and duration of immune suppression is not commonly quantified in either pre-clinical or clinical studies to date. This investigation highlights the potential for immune therapeutics that do not deplete or suppress the immune system in autoimmune disease. Overall, the therapeutic application for IgM-enriched therapeutics in T1D is an untapped avenue that holds great promise outside of its current clinical applications.

## Materials and methods

### Animals

C57BL/6 J (B6) and NOD/ShiLtJ (NOD) mice were purchased from The Jackson Laboratory (Bar Harbor, ME), Mice were housed in a specific-pathogen–free facility at Vanderbilt University. Female mice were used in this study as the penetrance of diabetes is much higher. No male mice were used in this study. Animal experiments were reviewed and approved by the Vanderbilt IACUC in keeping with ethical principles. The study is designed, performed, and reported in keeping with ARRIVE guidelines.

### Pentaglobin treatment and dosage

Pentaglobin was provided by Biotest AG (Dreieich, Germany); Biotest AG did not have any other contributions to the study design or data interpretation. For diabetes reversal, NOD mice were treated by i.p. injection with (300 μg) of Pentaglobin on day 1, 4, 7, and 10 after diabetes onset. For cellular analysis, NOD and B6 mice were treated by i.p. injection with an initial dose of 300 μg Pentaglobin on day 1 followed by 300-μg doses on days 4 and sacrificed on day 10.

### Flow cytometry and antibodies

Spleen and thymus were rendered to single-cell suspensions by crushing through a 70-μm filter. Splenocytes or thymocytes were stained with fluorophore-conjugated antibodies purchased from either BD Biosciences (San Jose, CA), eBioscience (San Diego, CA).

### Mouse-anti human antibody ELISA

25uls of murine serum was collected via submandibular bleed from each mouse before and after Pentaglobin treatment. The mouse anti-human antibody (MAHA) ELISA (Eagle Biosciences) was carried out according to manufacturer’s directions. It was read at 450 nm absorbance on a plate reader. Concentration of anti-human IgG was determined via standard curve, and validated via internal controls of know concentration provided by the manufacturer.

### Statistics

Statistical analysis was performed with Prism 5 software (GraphPad, La Jolla, CA) using the Mann–Whitney *U* test. One- or two-way ANOVA followed by Bonferroni posttest was used to compare multiple groups. Statistical comparisons with *P* ≤ 0.05 were deemed significant.

### Study approval

The institutional animal care and use committee at Vanderbilt University approved all procedures carried out during this study.

## Supplementary Information

Supplementary Figure 1.

Supplementary Figure 2.
