# Supplementary material for: A human IgM enriched immunoglobulin preparation, Pentaglobin, reverses autoimmune diabetes without immune suppression in NOD mice
Source: Sci Rep. 2022 Jul 11;12:11731. doi: 10.1038/s41598-022-15676-8 (PMC9274958; doi:10.1038/s41598-022-15676-8)
Supplement: Supplementary file 1 — Supplementary Figure 1. [file 41598_2022_15676_MOESM1_ESM.pdf]

Supplemental 1

A.

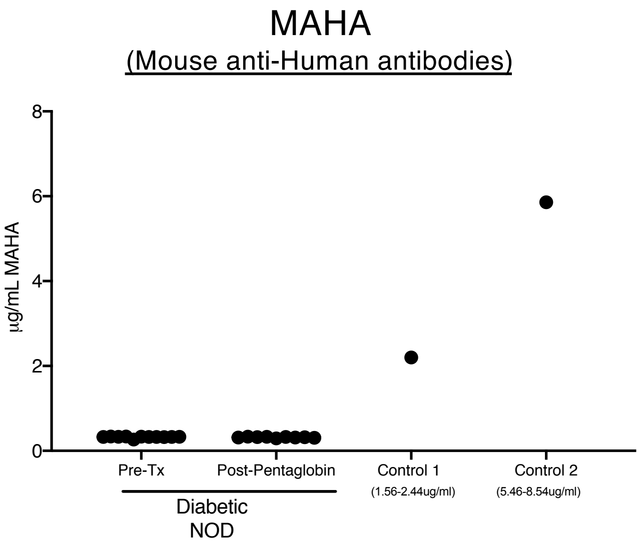

Supplemental 1. Pentaglobin does not induce an anti-human antibody response in diabetic NOD mouse. NOD mice that were treated with Pentaglobin were bled the day before their first dose and after the last dose of 300ug Pentaglobin. Their MAHA response was analyzed by ELISA. No anti-human response was detected before or after treatment in any mice assessed.
