# Supplementary material for: A human IgM enriched immunoglobulin preparation, Pentaglobin, reverses autoimmune diabetes without immune suppression in NOD mice
Source: Sci Rep. 2022 Jul 11;12:11731. doi: 10.1038/s41598-022-15676-8 (PMC9274958; doi:10.1038/s41598-022-15676-8)
Supplement: Supplementary file 2 — Supplementary Figure 2. [file 41598_2022_15676_MOESM2_ESM.pdf]

Supplemental 2

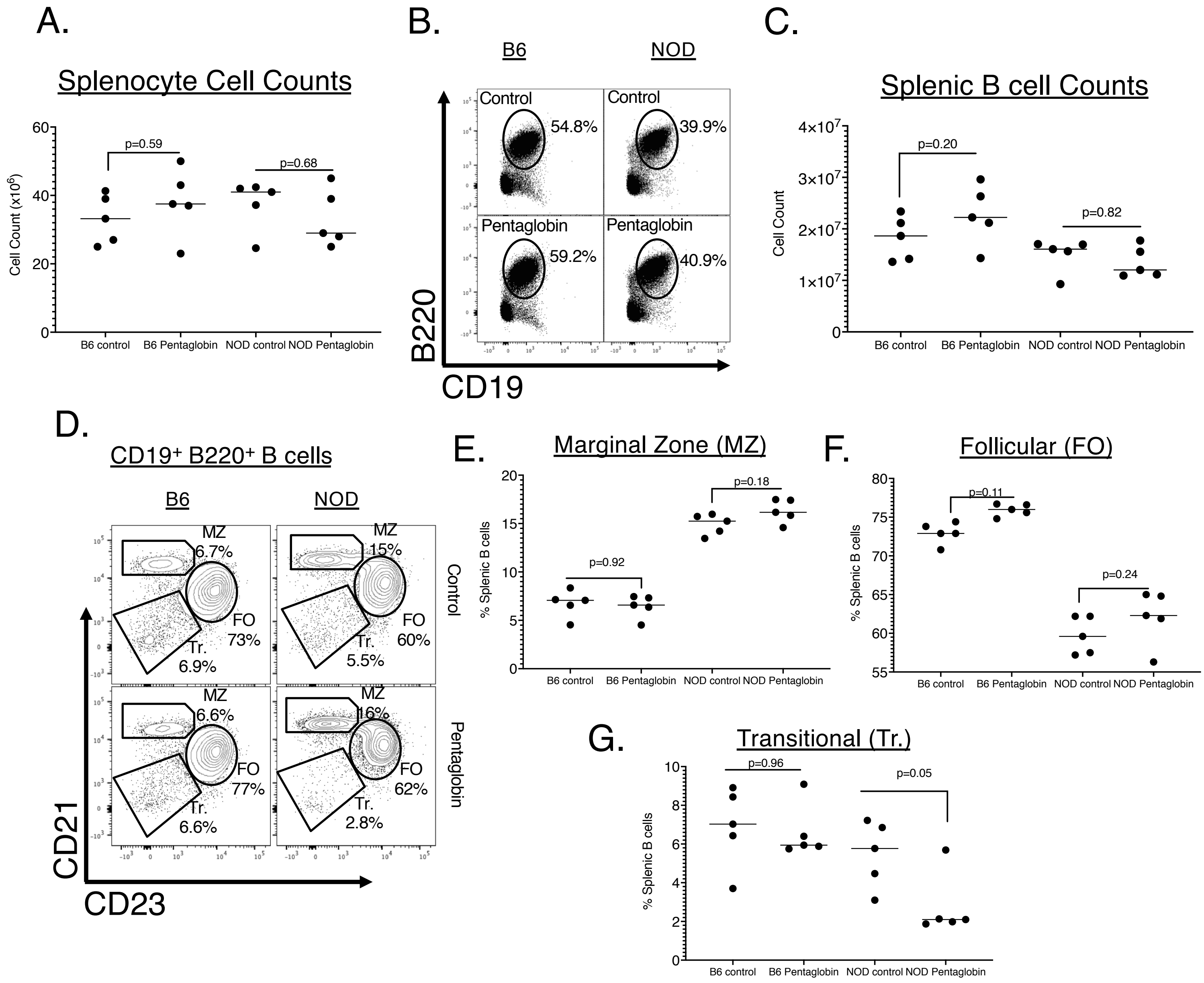

Supplemental 2. Pentaglobin does not robustly restore B cell homeostasis to the NOD mouse. A) Original studies indicated that healthy donor IgM induced expansion in total splenocyte numbers. This expansion of total splenocytes or B cells was not observed here **B**. Quantified in **C**. D) Using flow cytometry marginal zone (CD21hi CD23 negative) and follicular (CD21intermediate CD23+), and transitional (CD21lo and CD23 lo) were measured. NOD mice have expanded marginal zone B cells and reduced transitional zone B cells compared to B6 mice. Marginal zone B cells in NOD mice treated with Pentaglobin were not reduced. A decrease in the transitional B cells in NOD mice treated with Pentaglobin was observed. Little change was noted in the follicular zone B cells between any group. Percentages display graphically in **E-G**.
